# Clinical Decision Making for Intraoperative Auditory Brainstem Response Testing in Children following Tympanostomy Tube Placement

**DOI:** 10.3390/jcm12030830

**Published:** 2023-01-20

**Authors:** Maria Dietrich, Heike Schade, Jennifer Nadal, Sabine Keiner, Götz Schade

**Affiliations:** 1 Department of Psychiatry and Psychotherapy, University Hospital Bonn, Venusberg-Campus 1, 53127 Bonn, Germany; 2 Private Practice Kleinow, Pfarrer-Kenntemich-Platz 11, 53840 Troisdorf, Germany; 3 Department of Medical Biometrics, Informatics and Epidemiology, University Hospital Bonn, Venusberg-Campus 1, 53127 Bonn, Germany; 4 Department of Otorhinolaryngology—Head and Neck Surgery, University Hospital Bonn, Venusberg-Campus 1, 53127 Bonn, Germany; 5 Division of Phoniatrics and Pediatric Audiology, Department of Otorhinolaryngology—Head and Neck Surgery, University Hospital Bonn, Venusberg-Campus 1, 53127 Bonn, Germany

**Keywords:** auditory brainstem response, otitis media with effusion, tympanostomy tube, temporary threshold shift, myringotomy

## Abstract

Intraoperative auditory brainstem response (ioABR) testing following tympanostomy tube (TT) placement may be biased due to temporary threshold shifts (TTS). The purpose of the study was to assess the evidence for TTS in children who have undergone ioABR using prolonged latencies of wave I (males > 1.95 ms, females > 1.88 ms) as a marker of a persisting air–bone gap. Eighty-three children underwent ioABR following surgical procedures at University Hospital Bonn, Germany. The primary outcome measure was the latency of wave I at 80-dB SPL. The total sample consisted of 66 males (79.5%) and 17 females (20.5%) with a mean (SD) age of 46.4 (26.6) months. Of 163 operated ears (83 children), 72 (44.2%) had no middle ear fluid, 19 (11.6%) serous fluid, and 72 (44.2%) mucoid fluid. The risk of having a prolonged latency of wave I at 80-dB SPL was OR 4.61 (95% CI 2.01–10.59; *p* < 0.001) in those with mucoid fluid as compared to those without mucoid fluid. Intraoperative ABR results should account for sex differences and be interpreted with caution and be verified. Ultimately, parents should be engaged in a preoperative discussion to decide if an ioABR should be postponed if mucoid fluid was found.

## 1. Introduction

Auditory brainstem response (ABR) testing is an objective diagnostic tool [1,2,3] and considered the gold standard when reliable reporting of hearing thresholds is not possible [4]. An ABR must be conducted in melatonin-induced sleep or at minimum in “video sedation” since activity-based muscle potentials would mask the early auditory-evoked potentials (EAEP). Hence, one needs a calm and cooperative patient [5]. However, an ABR is often not feasible in children with poor compliance as well as those who are intellectually disabled. Yet, middle and inner ear hearing impairments are particularly common in children with Down syndrome [6]. For these children, an intraoperative ABR (ioABR) is imperative. The ioABR is preoperatively scheduled and personnel committed. In Germany, for example, ioABR testing is typically conducted even if a tympanostomy tube (TT) placement is indicated due to intraoperatively determined fluid.

However, several authors [7,8,9,10,11] pointed out that ioABR results post-TT insertion must be interpreted with caution. Results often indicate an elevated hearing threshold, which in many cases spontaneously improves within days to weeks. Among various reasons for the temporary threshold shift (TTS), proximal ones are the ongoing inflammatory mucosal changes in the middle ear as well as the acute noise trauma from suctioning of the middle ear effusion [7,12]. Mason et al. [9] showed in three case studies that in six of fourteen ears post-TT placement the threshold shift was 15 dB and more, but no threshold discrepancies were noted for ioABR in ears with no fluid determined post-myringotomy or when myringotomy was skipped due to well-ventilated middle ears. Richardson et al. [13] reported of difficulties in proving otoacoustic emission (OAE) in the operating room immediately following TT placement in comparison to normal ears (reduced OAE-responses in 31 of 80 and missing OAE-responses in 39 of 80 children).

Griffiths et al. [10] could determine preoperative transitory evoked otoacoustic emissions (TEOAE) in 30% of ears and immediately post-surgery only in 20%, yet after 6 weeks post-surgery in 82% of ears. In ten children (twenty ears), thirteen ears showed shifted hearing thresholds by as much as 20 dB and immediately post-TT insertion in the subsequent ioABR, and six ears showed even thresholds of 35–40 dB. Yorgason et al. in 2010 [8] recommended to always verify the ioABR threshold post-TT insertion with behavioral audiometric testing or OAE-testing (if needed ABR). Their results indicated that hearing thresholds often deviated between 10–20 dB, and in exceptional cases even up to 45 dB.

Dornan et al. [11] reported similar numbers. During testing there was a mean (SD) measurement difference between ioABR post-TT insertion and follow-up audiologic testing of 9.7 (12.7) dB with a single maximum discrepancy of 45 dB. Such measurement differences between intra- and post-operative hearing testing were lower, with a mean (SD) of 3.8 (8.6) dB in the case of no middle ear fluid based on myringotomy or in the case of good ventilation, so that a TT placement could be avoided.

Regarding time-to-normalization, a study by Boudewyns et al. [14] investigating otitis media with effusion in babies referred from universal newborn-hearing screenings also tested ABR thresholds post-TT insertion and found that 31% of children had abnormal thresholds. All children had normal hearing by a median age of 11.1 months.

Finally, a recent pilot study by Martinovic et al. [12] investigated twenty children (38 ears) who were scheduled for a TT insertion. ABR thresholds pre- and post-tube placement did not significantly differ between those with and without fluid. However, there was a trend that at least some children with serous and mucoid fluid may show worse ABR thresholds after tube insertion.

There is still a paucity of data to guide clinical practice with regard to ioABR measurements post-TT insertion. Therefore, the purpose of the study was to determine evidence for TTS based on the prolonged latencies of wave I (Jewett I = J I) in a large retrospective sample of children including those who have undergone ioABR following surgical procedures such as myringotomy and TT insertion. The ABR latency delay of wave I (J I) indexes with high accuracy the presence or absence of an otitis media with effusion [15] and can be used as a marker of a persisting air–bone gap. Wave I originates at the level of the distal auditory nerve [16]. An air–bone gap due to disturbed sound transmission in the middle ear would inevitably lead to a prolonged latency of wave I of the EAEP. Based on anecdotal evidence at University Hospital Bonn, many children who underwent ioABR post-TT placement presented with temporary thresholds on the range of 40–50 dB, triggering appointments for hearing aid fitting. However, the hearing threshold normalized by the time of the appointment. The hypothesis was that children with TT insertion due to fluid in the ear were at greater risk for a persisting air–bone gap than those without TT insertion.

## 2. Materials and Methods

The retrospective study included pediatric patients who between February 2008 and December 2013 underwent click ioABR at University Hospital Bonn in part with prior adenoidectomy, myringotomy, and if needed TT insertion. The Ethics Committee of the University of Bonn determined the study to be exempt from review. Age, height, and weight were recorded as well as the presence of adenoids (grade I no obstruction, II partial obstruction, or III complete obstruction), middle ear fluid (none, serous, mucoid), and surgical procedures such as adenoidectomies, myringotomies, and TT insertions. Intraoperative ABR latency times of wave I at 80-dB, or 90-dB and 100-dB SPL if needed, were collected as well as the latencies of waves III, V, and the interpeak latency I-V. Wave V (Jewett V = J V) correlates well with the actual hearing threshold and can be found approximately 0–10 dB below the threshold of detection [16]. Wave III and V latency delays may indicate maturation defects of the auditory pathway and retrocochlear disorders [17]. The ABR equipment used for this study was the ERA-System Corona (Pilot Blankenfelde medizinisch elektronische Geräte GmbH, Blankenfelde, Germany). The stimulus rate was 17.17 Hz. The number of sweeps per run was 1500. Experienced audiometric medical-technical assistants with 20–30 years of professional experience performed the assessment and interpretation with most assessments performed by one particular assistant.

In addition, pre- or post-operative notes about binocular-microscopic results and available audiometric data were collected (pure tone or free-field audiometry at 500 Hz, 1 kHz, 2 kHz, and 4 kHz; transitory evoked otoacoustic emission [TEOAE] and distortion product otoacoustic emission [DPOAE]; speech audiometry). Lastly, comorbidities and noteworthy family history, duration of surgery, anesthesia, and ABR examinations were documented. Total time of anesthesia refers to the sum of surgical procedures, which may include adenoidectomy, unilateral or bilateral myringotomy, unilateral or bilateral TT placement or change (or removal), as well as the additional ioABR. Follow-up audiometric tests typically took place at the referring otolaryngologist’s office.

The primary outcome measure was the latency of wave I. A latency of >1.95 ms was historically considered abnormal at 80-dB SPL at the study site, but significant sex differences in the control data set required a different cut-off for females (t = 2.72, *p* < 0.0091). We reviewed ABR data from 98 ears with normal tympanograms collected between January 2021 and September 2022 (50 children: 26 males, 24 females). The age ranged from 2 months to 89 months with a mean of 29.5 months (SD = 23.0). Wave I values for both ears combined had a mean of 1.67 (SD = 0.14) with a range from 1.21 to 1.90 ms. For male ears the values were a mean of 1.72 (SD = 0.12) with a range from 1.44 to 1.90; mean of 1.62 (SD = 0.14) with a range of 1.21 to 1.89 for female ears. Using the formula M + (1.95 × SD), the threshold for boys remains at 1.96 (>1.95) but is lower for girls at 1.89 (>1.88). If wave I could not be elicited at 80-dB SPL, then measurements were taken at 90-, 100-dB SPL. For testing with elevated sound pressure levels, a generally accepted norm value is missing. For this study, a threshold of 1.90 ms for the measurement of latency of wave I at 90-db SPL was chosen. Greater SPLs lead to faster conduction velocity and thus to a shorter latency.

Statistical analyses were performed using SPSS (IBM SPSS Statistics version 27). We describe the overall presence of mucoid fluid groups in the study population using mean values and standard deviations for normally distributed variables, and median values and interquartile ranges for non-normally distributed variables. The values of categorical variables are presented as frequency distributions with percentages. For the outcome presence of prolonged latencies, a mixed-effects model was used to account for the hierarchical nature of the data (ears nested in patient), including mucoid fluid or fluid in the ear as covariables. The resulting odds ratios (ORs) are presented with 95% confidence intervals. A two-sided *p* value of <0.05 was considered significant.

## 3. Results

The initial study sample included 95 consecutively enrolled children between 4 and 202 months (median 42) who between February 2008 and December 2013 underwent click ioABR at University Hospital Bonn in part with prior adenoidectomy, myringotomy, and, if needed, TT insertion. Of those, 83 children between 4 and 134 months have undergone an ioABR post-unilateral or bilateral myringotomy with subsequent TT insertion as needed and are the focus of the study. There were 66 males with a mean age of 47.6 months (SD = 27.0, range 4 to 134 months) and 17 females with a mean age of 42.1 months (SD = 25.3, range 7 to 106 months. The median age was similar, with 42 for males and 40 for females. Table 1 summarizes the characteristics of the total study sample and subsamples based on the presence or absence of mucoid fluid.

**Figure 1:**
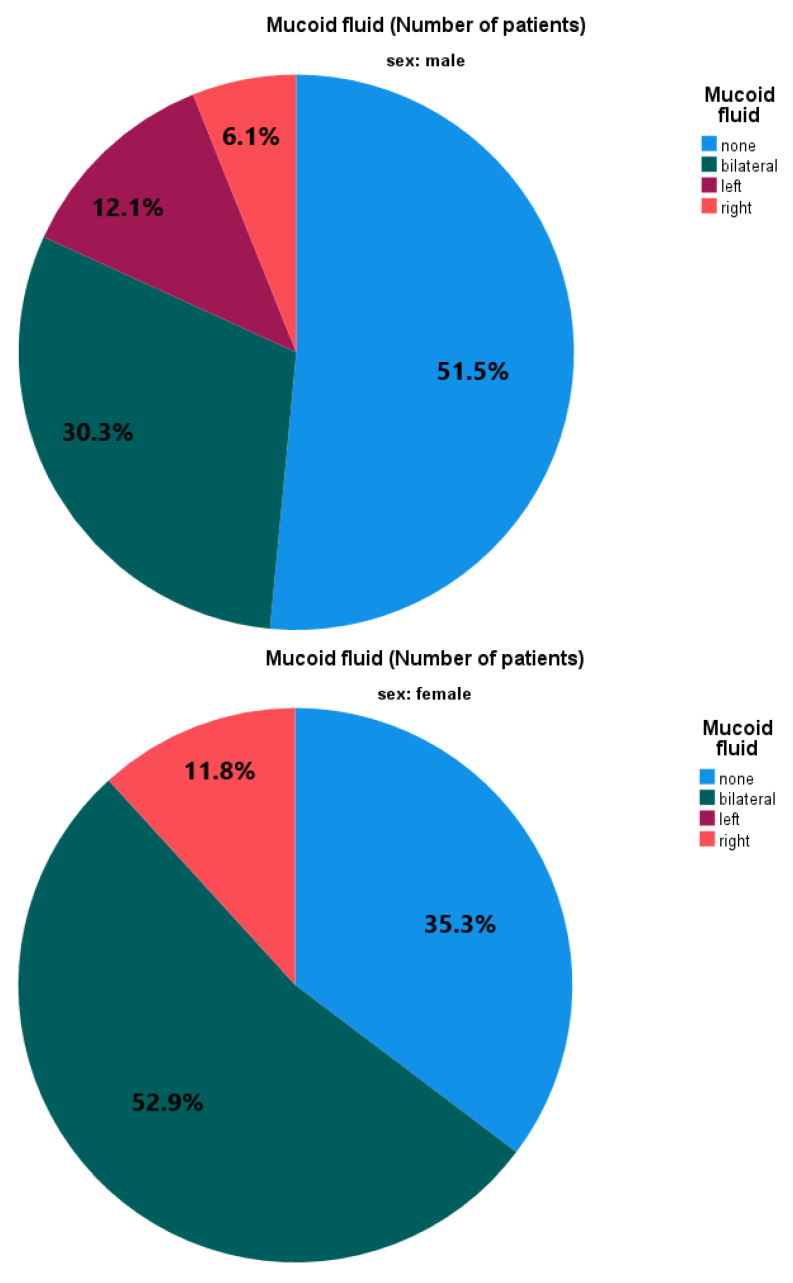
Absence and presence of otitis media with mucoid effusion by sex and laterality.

Of 163 operated ears, 72 (44.2%) had no fluid, 19 (11.6%) serous fluid, and 72 (44.2%) mucoid fluid. Slightly over half of the patients (n = 43, 51.8%) had mucoid fluid compared to no mucoid fluid, i.e., no fluid or serous fluid. Of those, twenty-nine had bilateral, seven left, and seven right mucoid fluid. Figure 1 illustrates the absence or presence of otitis media with and without mucoid effusion by sex and laterality.

Descriptive data of operative procedures by effusion type are shown in Table 2. A single myringotomy (without TT insertion) occurred in 37 left and 38 right ears and TT insertion post-myringotomy in 40 left and 38 right ears. TT change occurred once on the left and four times on the right side. Three TTs were already in place, one was removed, and for one ear the corresponding notes are missing. In the presence of mucoid fluid, a single myringotomy never occurred on the left side but occurred once on the right side. Regarding TT insertion in those with mucoid effusion, 35 ears underwent TT insertion on the left side and 32 ears on the right side. A TT change occurred once on the right side.

**Figure 2:**
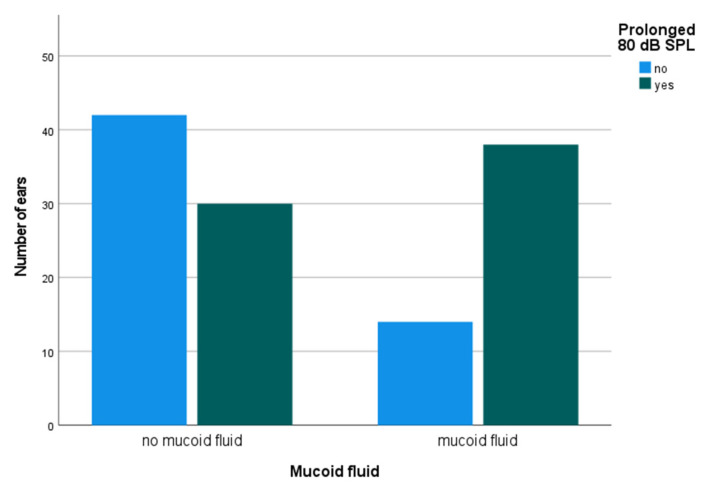
Prolonged latency of wave I (males > 1.95 ms, females > 1.88 ms) at 80-dB SPL during intraoperative auditory brainstem response testing comparing ears with and without mucoid fluid (n = 124 ears).

Latencies of wave I were measured in 83 children and can be found in Table 2. Wave I at 80-dB SPL was measured in 124 ears. The latencies ranged from 1.47 to 3.26 ms (mean [SD] 2.04 [0.38] ms). For 34 examined left ears without mucoid fluid (i.e., none or serous fluid), the mean (SD) was 1.91 (0.34) ms. For 29 left ears, where minutes prior an effusion was suctioned followed by a TT insertion, the mean (SD) was 2.18 (0.37) ms. The corresponding results (mean [SD]) for the right ears were 1.94 (0.33) ms for 38 ears without mucoid fluid and 2.22 (0.39) ms for 23 treated ears with mucoid fluid immediately prior to ioABR testing. Figure 2 displays the results in reference to normal and abnormal latencies by presence or absence of mucoid fluid.

In both groups, with and without mucoid fluid, there were children with prolonged latency of wave I at 80-dB SPL. In children without mucoid fluid, the prolonged latency of wave I could only be determined in 21 of 72 ears (29.2%), whereas in the group of children with mucoid fluid it could be determined in 38 of 52 ears (73.1%). As a function of sex, the percentage of male ears (n = 101) with mucoid fluid and prolonged latency of wave I at 80-dB SPL was 28.7 % (no mucoid and prolonged, 23.8%). Respective data for female ears (n = 23) were 39.1 % and 26.1 %.

For children tested at 90-dB SPL (n = 8), the corresponding results to testing at 80-dB SPL were as follows. Patients with mucoid fluid had a mean (SD) latency of 2.05 (0.34) ms on the left side and 2.19 (0.44) ms on the right side, while patients without mucoid fluid had latencies of 1.69 (0.06) ms on the left side and 1.87 (0.67) ms on the right side. However, this effect did not persist at the 100-dB SPL testing level. Seven children at this SPL had mucoid fluid with a mean (SD) latency of 1.61 (0.96) ms on the left side and 2.12 (0.32) ms on the right side. Without the presence of mucoid fluid, the value was 2.13 (0.45) ms on the left and 2.24 (0.53) ms on the right side.

When the statistical model was tested for wave I values at 80 dB only, the results were as follows. The risk of having a prolonged latency of wave I was OR 4.61 [95% CI 2.01–10.59] in those with mucoid fluid as compared to those without mucoid fluid (*p* < 0.001). The risk increased to OR 6.92 [95% CI 3.02–15.86] in those with fluid in the ear (serous or mucoid) as compared to those without fluid (*p* < 0.001).

**Figure 3:**
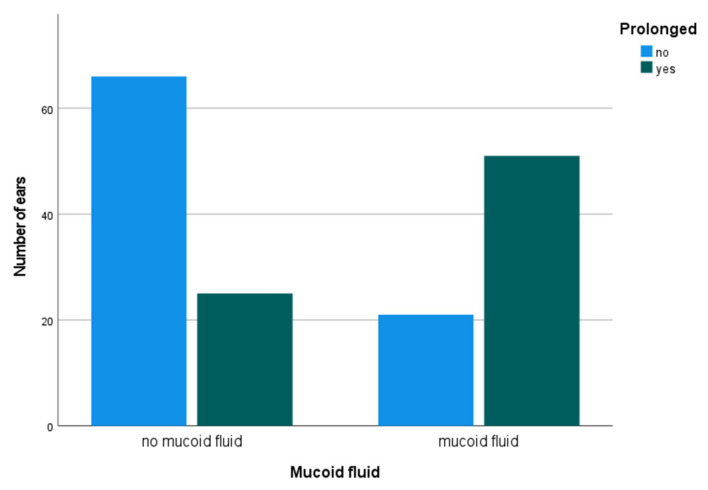
Prolonged intraoperative auditory brainstem response latencies of wave I across 80-, 90-, and 100-dB SPL comparing 153 ears with and without mucoid fluid (at 80 dB prolonged males > 1.95 ms and females > 1.88 ms; at 90 and 100 dB prolonged > 1.90 ms).

Across all testing levels for wave I (80-, 90-, and 100-dB SPL), the risk of having a prolonged latency of wave I was OR 8.24 [95% CI 4.01–16.92] in those with mucoid fluid as compared to those without mucoid fluid (*p* < 0.001). The risk was OR 7.61 [95% CI 3.67–15.79] in those with fluid in the ear as compared to those without fluid (*p* < 0.001). Figure 3 shows pooled results from all three testing levels. As a function of sex, the percentage of male ears (n = 129) with mucoid fluid and prolonged latency of wave I was 27.9% (no mucoid and prolonged, 14.0%). Respective data for female ears (n = 34) were 44.1% and 20.6%.

## 4. Discussion

The purpose of the study was to determine evidence for TTS based on prolonged latencies of wave I in a large retrospective sample of children including those who have undergone ioABR following TT insertion. The statistical results confirmed the hypothesis that ioABR results can be biased when obtained immediately post-TT insertion. Most patients who underwent TT insertion showed postoperatively prolonged latencies of wave I (at 80-dB SPL), which was on average greater in children with mucoid fluid compared with children with serous and mucoid fluid combined (left, 2.18 ms vs. 2.16 ms; right, 2.22 ms vs. 2.17 ms) and more pronounced in contrast to measurements at 80, 90, and 100 dB combined. In direct comparison of 29 left and 23 right ears with mucoid fluid to 27 left and 27 right ears without effusion, there was still a difference by way of latency differences of wave I at 80 dB on the left with 2.18 ms vs. 1.86 ms, and on the right with 2.22 ms vs. 1.89 ms.

Age and sex differences in latencies of waves I, III, and V in ABR responses in normal hearing participants have been reported with latencies of waves I and V linearly increasing with age in men [18]. Data for children were sparse in that study. We used our own control data set to test for sex differences, which led to a lower cut-off for a prolonged wave I at 80 dB in females (>1.88 ms) than males (>1.95 ms), which was factored into our results.

As in other studies, not all patients with surgically treated mucoid fluid showed post-operatively prolonged latency. Five patients with bilateral mucoid fluid (i.e., 10 ears) and without abnormal latencies in ioABR did have a normal hearing threshold of 10–20 dB based on the analysis of wave V potentials. Two patients with bilateral mucoid fluid and unilateral latency increases had interaural latency differences of 0.51 up to 1.47 ms, indicating that only one side had abnormal conduction. In three patients who showed a unilateral latency increase, the contralateral side could not be tested, because one child had a unilateral auricular deformation and auditory canal malformation and in two patients the hearing threshold up to 100-dB SPL could not be determined and thus, the potential of wave I up to 100 dB.

An additional child with only a unilaterally abnormal latency post-TT insertion due to bilateral mucoid fluid also only had, unilaterally, a pathologic hearing threshold of 40–50 dB on the side with the increased latency, while the contralateral side was already at physiological 20–30 dB. In two patients with normal latencies, thresholds were 50–60 dB and 30–40 dB, respectively. A postoperative follow-up hearing test was unfortunately not available due to follow-up care in private practice. However, in one additional child with bilateral mucoid fluid, a unilateral latency delay, and a threshold of 30–40 dB, there was a bilateral normal result in follow-up OAE testing (TEOAE bilateral pass). In 18 of our patients with a TT insertion for bilateral mucoid fluid, there were still bilateral prolonged latencies of wave I during ioABR.

The data on surgical and total anesthesia time show that the average duration of surgery was 10 min., while the total duration of anesthesia (surgery plus ioABR) was close to two hours. Thus, children were exposed to an average of a more-than-10-fold duration of anesthesia because of subsequent ioABR. Yet, the ioABR measurement may be biased due to persisting prolonged latency, and thus may indicate a TTS with poorer hearing acuity than the actual expected inner ear performance of these children. In fact, the updated clinical practice guideline for TTs briefly acknowledges the problem and recommends the shared decision making of stakeholders [19], which aligns with the following recommendations based on the current data. If hearing thresholds barely indicate hearing aid fitting, the audiologist must be advised that the amplification of hearing aids is only adjusted due to a probably temporary air–bone gap. Thus, it would be desirable in such cases to refrain from ioABR testing immediately post-TT insertion and to conduct the ioABR a few weeks later or to conduct hearing testing with behavioral audiometric testing, OAE measurement, or ABR in melatonin-induced sedation if feasible. Such clinical decision making provides the audiologist with a reliable foundation for optimal hearing aid fitting. Ultimately, parents should be engaged in a preoperative discussion with the physician to decide if an ioABR should be postponed if mucoid middle ear fluid was found during surgery, which would require a TT placement. Consequently, the ioABR should only be performed in the same surgical session as the TT placement when parents, despite medical advice for a two-step procedure, wish to pursue or anesthesiologists advise against another anesthesia in the following weeks.

The study is limited by its retrospective nature. Unfortunately, most follow-up audiometric testing occurred off-site, so that it could not be determined with certainty in what time frame the thresholds returned to normal values. Bone-conduction testing was not included to help with the interpretation of the data. Other literature also recommended reporting anesthetic agents and other electronic equipment in the operating room [12].

## 5. Conclusions

Intraoperative ABR results can be biased when obtained immediately post-TT insertion. Most patients who underwent TT insertion showed postoperatively prolonged latencies of wave I (at 80-dB SPL), which was on average greater in children with mucoid fluid compared with children with serous and mucoid fluid combined. The surgeon should inform parents accordingly and devise an individual plan.

## Figures and Tables

**Table 1:** Participant characteristics (in percentages and mean and standard deviations).

		SUBTOTAL Presence of Mucoid Fluid			
	TOTAL	Bilateral	Left	Right	None
	n = 83	n = 29	n = 8	n = 6	n = 40
Sex					
male	66 (79.5%)	20 (24.1%)	8 (9.6%)	4 (4.8%)	34 (41.0%)
female	17 (20.5%)	9 (10.8%)	0	2 (2.4%)	6 (7.2%)
Age (months)	46.4 (26.6)	39.6 (19.7)	35.5 (14.7)	29.3 (26.6)	56.2 (29.6)
Weight (kg)	16.1 (5.4)	13.9 (3.9)	12.2 (1.7)	13.0 (5.3)	18.9 (5.7)
Height (cm)	100.1 (16.8)	94.0 (11.1)	92.4 (16.0)	88.7 (22.1)	107.0 (17.6)
Intellectual disability					
yes	31 (37.3%)	9 (10.6%)	3 (3.6%)	1 (1.2%)	18 (21.7%)
Adenoidectomy					
yes	36 (43.6%)	17 (20.5%)	4 (4.8%)	2 (2.4%)	13 (15.7%)
Adenoids I°	13 (33.3%)	2 (5.1%)	2 (5.1%)	1 (2.6%)	8 (20.5%)
Adenoids II°	20 (51.3%)	9 (23.1%)	1 (2.6%)	2 (5.1%)	8 (20.5%)
Adenoids III°	6 (15.4%)	4 (10.3%)	1 (2.6%)	0	1 (2.6%)
Anesthesia (min)	106.0 (16.8)	108.1 (19.0)	88.3 (20.7)	105.0 (10.8)	107.7 (29.9)
Time in OP (min)	12.2 (7.9)	13.3 (9.5)	11.4 (8.5)	12.8 (3.2)	11.3 (6.9)
ioABR (min)	63.5 (19.7)	67.8 (19.3)	45.5 (19.4)	55.0 (21.2)	64.5 (18.6)

Note. OP = operating room; ioABR = intraoperative ABR; adenoid grades, I no obstruction, II partial obstruction, III complete obstruction.

**Table 2:** Summary of effusion type, operative procedure, and intraoperative ABR findings (latencies in mean and standard deviation).

	TOTAL	Ears without Mucoid Fluid	Ears with Mucoid Fluid	Ears without Fluid	Ears with Fluid (Serous/Mucoid)
Single myringotomy	75 (46.0%)	74 (47.4%)	1 (0.6%)	53 (34.0%)	22 (14.1%)
TT placement post-myringotomy	76 (46.5%)	9 (5.8%)	67 (42.9%)	4 (2.6%)	72 (46.2%)
TT change	5 (3.1%)	4 (2.6%)	1 (0.6%)	4 (2.6%)	1 (0.6%)
Total	156 (95.7%)	87 (55.8%)	69 (44.2%)	61 (39.1%)	95 (60.9%)
Wave I 80-dB SPL (ms)	2.04 (0.38)	1.93 (0.33)	2.19 (0.38)	1.86 (0.29)	2.17 (0.38)
male	2.02 (0.37)	1.91 (0.33)	2.19 (0.36)	1.87 (0.30)	2.15 (0.37)
female	2.12 (0.42)	2.00 (0.37)	2.25 (0.45)	1.86 (0.25)	2.26 (0.43)
Wave I 90-dB SPL (ms)	2.02 (0.44)	1.80 (0.48)	2.14 (0.39)	1.80 (0.48)	2.14 (0.39)
male	1.96 (0.51)	1.80 (0.48)	2.11 (0.55)	1.80 (0.48)	2.11 (0.55)
female	2.18 (0.08)		2.18 (0.08)		2.17 (0.08)
Wave I 100-dB SPL (ms)	2.00 (0.59)	2.17 (0.42)	1.91 (0.66)	2.61	1.96 (0.58)
male	2.01 (0.73)	2.20 (0.38)	1.92 (0.87)	2.61	1.94 (0.74)
female	1.98 (0.34)	2.14 (0.63)	1.90 (0.19)		1.98 (0.34)
Wave III 80-dB SPL (ms)	4.34 (0.49)	4.25 (0.46)	4.47 (0.49)	4.16 (0.38)	4.48 (0.51)
male	4.35 (0.49)	4.27 (0.47)	4.48 (0.50)	4.17 (0.40)	4.50 (0.51)
female	4.29 (0.46)	4.17 (0.41)	4.43 (0.50)	4.06 (0.25)	4.44 (0.51)
Wave III 90-dB SPL (ms)	4.45 (0.45)	4.25 (0.52)	4.57 (0.38)	4.25 (0.52)	4.57 (0.38)
male	4.49 (0.53)	4.25 (0.52)	4.74 (0.45)	4.25 (0.52)	4.74 (0.45)
female	4.36 (0.08)		4.36 (0.08)		4.36 (0.08)
Wave III 100-dB SPL (ms)	4.24 (1.11)	4.43 (0.37)	4.14 (1.36)	4.90	4.20 (1.14)
male	4.16 (1.44)	4.53 (0.34)	3.97 (1.77)	4.90	4.07 (1.51)
female	4.37 (0.38)	4.29 (0.40)	4.41 (0.43)		4.37 (0.38)
Wave V 80-dB SPL (ms)	6.42 (0.55)	6.25 (0.49)	6.64 (0.55)	6.15 (0.76)	6.62 (0.59)
male	6.40 (0.52)	6.26 (0.49)	6.61 (0.51)	6.17 (0.36)	6.59 (0.57)
female	6.51 (0.65)	6.23 (0.50)	6.74 (0.68)	6.01 (0.24)	6.72 (0.66)
Wave V 90-dB SPL (ms)	6.76 (0.76)	6.45 (0.70)	6.93 (0.76)	6.27 (0.76)	6.92 (0.70)
male	6.83 (0.86)	6.35 (0.71)	7.18 (0.83)	6.27 (0.76)	7.13 (0.79)
female	6.56 (0.29)	7.05	6.44 (0.11)		6.56 (0.29)
Wave V 100-dB SPL (ms)	6.04 (1.70)	6.42 (0.27)	5.85 (2.07)	6.82	5.99 (1.74)
male	5.91 (2.22)	6.45 (0.33)	5.65 (2.75)	6.82	5.80 (2.34)
female	6.24 (0.26)	6.40 (0.28)	6.17 (0.25)		6.24 (0.26)
ioABR (all wave I)					
Prolonged	76 (46.6%)	25 (15.3%)	51 (31.3%)	14 (8.6%)	62 (36.0%)
Not prolonged	87 (53.4%)	66 (40.5%)	21 (12.9%)	51 (31.3%)	36 (22.1%)

Note. TT = tympanostomy tube; ioABR = intraoperative ABR.

## Data Availability

The data presented in this study are available on request from the corresponding author.
